# Supplementary material for: Analyses of cancer incidence and other morbidities in gamma irradiated B6CF1 mice
Source: PLoS One. 2020 Aug 20;15(8):e0231510. doi: 10.1371/journal.pone.0231510 (PMC7440931; doi:10.1371/journal.pone.0231510)
Supplement: S1 File — (DOCX) [file pone.0231510.s001.docx]

**Supplemental methods:**

**Robustness testing for controls analysis:**

The models below were used for the control analysis robustness testing performed for **Supplemental Table 2**:

$\lambda\left( t \right)_{sex}= \lambda_{0, sex}\left( t \right)e^{\left( \beta_{1,sex}experiment \right)}$ (D) stratified by sex

$\lambda\left( t \right)_{sex}= \lambda_{0, sex}\left( t \right)e^{\left( \beta_{1,sex}fractions \right)}$ (E) stratified by sex

$\lambda\left( t \right)_{sex}= \lambda_{0, sex}\left( t \right)e^{\left( \beta_{1,sex}first irrad \right)}$ (F) stratified by sex

$\lambda\left( t \right)= \lambda_{0}\left( t \right)e^{\left( \beta_{1}sex+\beta_{2}fractions(1 \right)+\beta_{3}fractions\left( 24 \right)+ \beta_{4}fractions\left( 60 \right)+ \beta_{5}fractions(120)}$ (G) treated fractions as categorical variable

$\lambda\left( t \right)_{sex}= \lambda_{0, sex}\left( t \right)e^{\left( \beta_{1sex}fractions\left( 1 \right)+ \beta_{2}fractions\left( 24 \right)+\beta_{3}fractions\left( 60 \right)+ \beta_{4}fractions\left( 120 \right) \right)}$ (H) stratified by sex and treated fractions as categorical variable

**Robustness tests for analysis of gamma irradiated mice:**

The main survival model for gamma irradiated mice used fractions and age first irradiated as predictors, but we listed “NA” for these values for sham irradiated mice. We hypothesized that mice treated with more fractions and mice that were first irradiated later in life would both have an increase survival probability. An issue arises when treating these predictors as continuous variables. The control mice would have shown an opposite trend from the irradiated mice, potentially causing difficulty when fitting the dose response model. Because we were concerned with the dose response, we used NA for control mice, but included true values for age first irradiated and total number of fractions as part of our robustness testing. Below is a full list of models used for the robustness tests that were performed for **Supplemental Figure 3**.

3B $\lambda\left( t \right)= \lambda_{0}\left( t \right)e^{\left( \beta_{1}sex+\beta_{2}first irrad + \beta_{3}total dose+ \beta_{4}fractions+\beta_{5}total dose:fractions \right)}$, did not exclude mice first irradiated > 500 days

3D

$\lambda\left( t \right)_{sex}= \lambda_{0, sex}\left( t \right)e^{\left( \beta_{1,sex}first irrad + \beta_{2,sex}total dose + \beta_{3,sex}fractionated+\beta_{4,sex}total dose:fractions \right)}$, stratified by sex

3F

$\lambda\left( t \right)= \lambda_{0}\left( t \right)e^{\left( \begin{aligned} \beta_{1}sex+\beta_{2}first irrad + \beta_{3}total dose+ \beta_{4}fractions\left( 24 \right)+ \beta_{5}fractions\left( 60 \right)+ \beta_{6}fractions\left( 120 \right)+\beta_{7}total dose:fractions\left( 24 \right) \\ + \beta_{8}total dose:fractions\left( 60 \right)+ \beta_{9}total dose:fractions(120) \end{aligned} \right)}$, treated fractions as a categorical variable

3H

$\lambda\left( t \right)= \lambda_{0}\left( t \right)e^{\left( \beta_{1}sex+\beta_{2}first irrad + \beta_{3}total dose+ \beta_{4}fractions+\beta_{5}total dose:fractions \right)}$, included true values for age first irradiated and number of fractions for mice that received a total dose of 0Gy

3J

$\lambda\left( t \right)= \lambda_{0}\left( t \right)e^{\left( \beta_{1}sex+\beta_{2}first irrad + \beta_{3}total dose+ \beta_{4}fractions+\beta_{5}total dose:fractions \right)}$, did not exclude mice with total dose given in 300 fractions

3L

$\lambda\left( t \right)= \lambda_{0}\left( t \right)e^{\left( \begin{aligned} \beta_{1}sex+\beta_{2}first irrad + \beta_{3}total dose+ \beta_{4}fractions\left( 24 \right)+ \beta_{5}fractions\left( 60 \right)+ \beta_{6}fractions\left( 120 \right)+\beta_{7}total dose:fractions\left( 24 \right) \\ + \beta_{8}total dose:fractions\left( 60 \right)+ \beta_{9}total dose:fractions(120) \end{aligned} \right)}$, did not exclude mice with total dose given in 300 fractions and treated fractions as a categorical variable
