# Supplementary material for: Analyses of cancer incidence and other morbidities in gamma irradiated B6CF1 mice
Source: PLoS One. 2020 Aug 20;15(8):e0231510. doi: 10.1371/journal.pone.0231510 (PMC7440931; doi:10.1371/journal.pone.0231510)
Supplement: S2 File — (PDF) [file pone.0231510.s002.pdf]

Supplementary figures and tables

Analyses of cancer incidence and other morbidities in gamma irradiated B6CF1 mice.

Alia Zander[1], Tatjana Paunesku[1], Gayle Woloschak[1]

[1]Northwestern University, Feinberg School of Medicine, Radiation Oncology, Chicago, IL

List of supplementary tables and figures

- Table S1: censored mice
- Table S2: filtered mice
- Table S3: robustness test for controls analysis
- Table S4: Janus codes to match IES codes
- Table S5: Janus conditions for mice used in IES comparison
- Table S6: control mice including 300 fractions – robustness tests
- Figure S1: controls analysis
- Figure S2: 300 fractions model (continuous + KM models, KM curve by COD)
- Figure S3: Robustness testing for Cox PH model with gamma irradiated mice
- Figure S4: KM curves for gamma irradiated mice
- Figure S5: Cause specific hazards – Lung tumors, non-thymic lymphoma, tumors minus lung
- Figure S6: CIF with 6Gy dose limit

Supplementary Table S1: Cause of Death (COD) codes for censored mice and sample size for each group

Supplementary Table S1: Cause of Death (COD) codes for censored mice and the number of mice in each group.

Mice Censored	# of mice
COD – Accidental death	47
COD – Escaped during irradiation	8
COD - Discarded	207
COD – Improper irradiation	77
COD - Missing	29
COD – Sacrifice, programmed	19
No lethal disease listed	936

Supplementary Table S2: Data filtering for gamma analysis

Supplementary Table S2: Description of which mice were removed from our analysis, the corresponding reasoning, and the resulting sample size. N=4330 for control mice and N=7848 for gamma irradiated mice.

Data removed	Reasoning	# of mice
-	-	50110
JM11	Not a true data set	49,225
JM10	Different species – Peromyscus leucopus	46,835
JM14 mice treated with radioprotectors	Beyond the scope of our project	46,635
Breeder mice	Held under different conditions	43,428
JM2 mice	Held under different conditions	31,843
COD – removal to another experiment	Mice listed under different experiment, do not want to double count	28,153
JM12 mice	Controls analysis showed significant difference	27,553
JM3 mice	Controls analysis showed significant difference	24,478
Mice irradiated with 300 fractions	Controls analysis showed significant difference	23,953
JM8 mice	Experimental design – separate analysis required	22,626
Neutron irradiated mice	Different radiation quality - separate analysis	12178

Supplementary Figure S1: Cox PH model predicted graphs and table output for controls after filtering

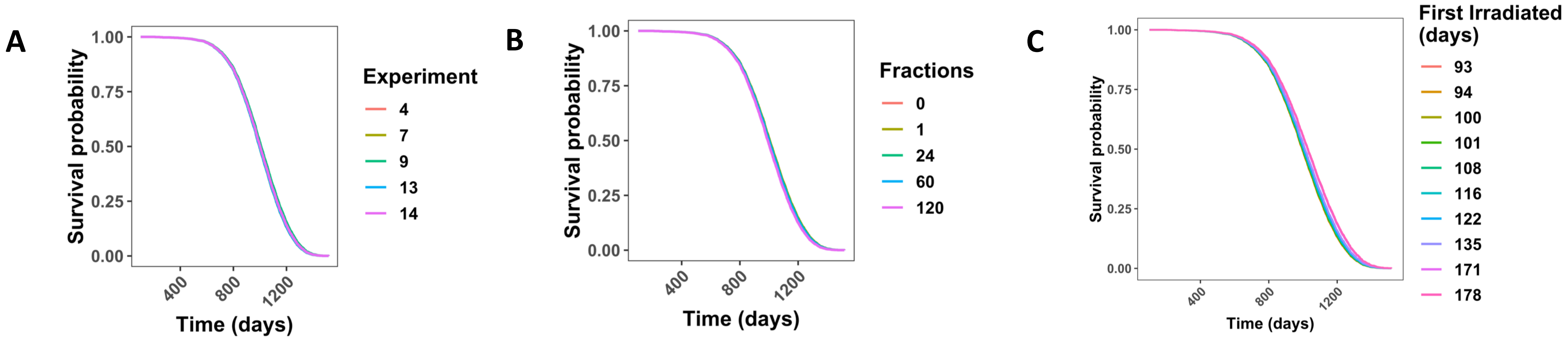

D

Model Group	Independent Variable	Parameter Estimate	Hazard Ratio (95% CI)	P-value
A	Sex	-0.119	0.888 (0.83, 0.949)	**0.001**
A	Expt 7	0.025	1.025 (0.914, 1.15)	0.67
A	Expt 9	-0.057	0.945 (0.863, 1.035)	0.225
A	Expt 13	0.02	1.021 (0.932, 1.118)	0.659
A	Expt 14	-0.009	0.991 (0.877, 1.12)	0.884
B	Sex	-0.11	0.896 (0.839, 0.957)	**0.001**
B	Fractions	0.001	1.0007 (1, 1.002)	0.17
C	Sex	-0.096	0.908 (0.85, 0.96)	**0.003**
C	First Irradiated	-0.002	0.998 (0.995, 1.001)	0.113

Supplementary Figure S1: Cox proportional hazard (PH) survival curves produced using all data filtered by the criteria in **Table 1** that also had a total dose of 0Gy. Age at death was used as a time scale with sex and **(A)** experiment, **(B)** number of fractions, or **(C)** the age first irradiated as independent variables. The predicted outcomes shown are for female mice. Parameter estimates and p-values from each model are shown in **D**. Kaplan Meier curves with **(E)** sex, **(F)** number of fractions, **(G)** age first irradiated, and **(H)** experiment group were evaluated to test the proportional hazards assumption of these models.

Supplementary Figure S1: KM curves for control mice to validate Cox PH model

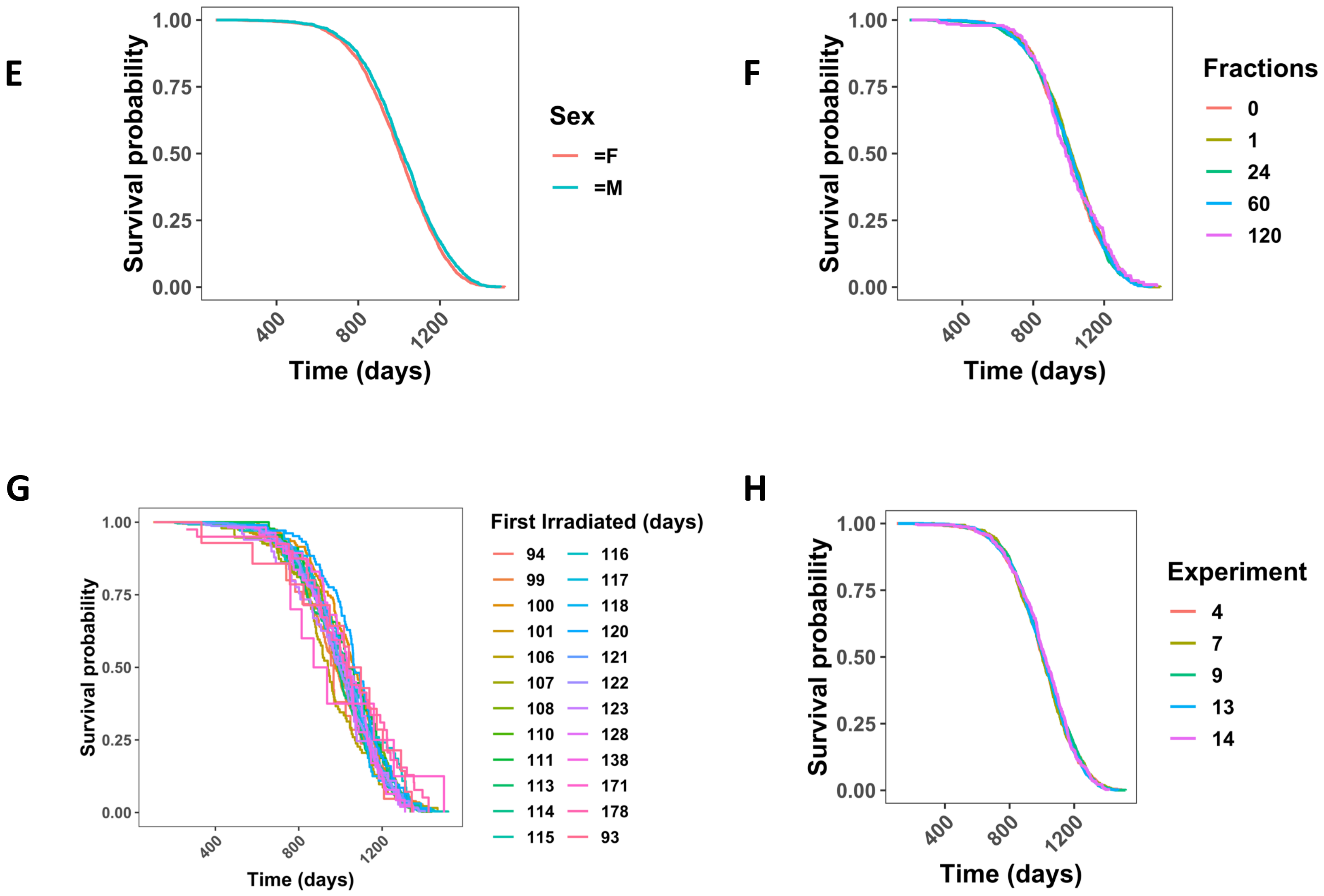

Supplementary Figure S1: Cox proportional hazard (PH) survival curves produced using all data filtered by the criteria in **Table 1** that also had a total dose of 0Gy. Age at death was used as a time scale with sex and **(A)** experiment, **(B)** number of fractions, or **(C)** the age first irradiated as independent variables. Parameter estimates and p-values from each model are shown in **D**. Kaplan Meier curves with **(E)** sex, **(F)** number of fractions, **(G)** age first irradiated, and **(H)** experiment group were evaluated to test the proportional hazards assumption of these models.

Supplementary Table S3: Robustness analysis Cox PH Controls (supplemental methods shows models used for each)

Supplementary Table S3: Robustness tests for Cox Proportional Hazards models for controls analysis.

Model Group	Independent Variable (reference)	Parameter Estimate	Hazard Ratio (95% CI)	P
D	Sex - stratify	NA	NA	NA
D	Expt 7 (Expt 4)	0.027	1.027 (0.92, 1.15)	0.645
D	Expt 9 (Expt 4)	-0.056	0.945 (0.86, 1.04)	0.230
D	Expt 13 (Expt 4)	0.0229	1.023 (0.93, 1.12)	0.621
D	Expt 14 (Expt 4)	-0.010	0.990 (0.88, 1.12)	0.876
E	Sex - stratify	NA	NA	NA
E	Fractions	0.001	1.001 (1.00, 1.002)	0.158
F	Sex - stratify	NA	NA	NA
F	First Irradiated	-0.002	0.998 (0.995, 1)	0.099
G	Sex	-0.114	0.892 (0.83, 0.95)	**0.001**
G	Fractions 1 (Fractions 0)	-0.083	0.921 (0.83, 1.03)	0.133
G	Fractions 24 (Fractions 0)	-0.014	0.986 (0.88, 1.11)	0.808
G	Fractions 60 (Fractions 0)	-0.004	0.996 (0.89, 1.11)	0.941
G	Fractions 120 (Fractions 0)	-0.005	0.995 (0.84, 1.18)	0.955
H	Sex - stratify	NA	NA	NA
H	Fractions 1 (Fractions 0)	-0.084	0.919 (0.83, 1.024)	0.126
H	Fractions 24 (Fractions 0)	-0.017	0.983 (0.87, 1.11)	0.772
H	Fractions 60 (Fractions 0)	-0.004	0.996 (0.89, 1.11)	0.947
H	Fractions 120 (Fractions 0)	-0.005	0.995 (0.84, 1.19)	0.956

Supplementary Table S4: Grouping B6CF1 Janus COD codes to match B6C3F1 IES categories

Supplementary table S4: Grouping B6CF1 Janus cause of death codes to match B6C3F1 IES categories

New code name:	Old code name(s): decoded (coded)
Circulatory	Vascular (TVAS), Heart (THRT), Spleen (TSPL)
Digestive	Caecum (TCEC), Colon (TCOL), Duodenum (TDUO), Esophagus (TESO), Ileum (TILE), Jejunum (TJEJ), Miscellaneous digestive system (TMID), Pancreas (TPAN), Salivary gland (TSGL), Stomach (TSTO), Tongue (TTGE), Gallbladder (TGBL), Liver (TLIV)
Endocrine	Adrenal (TADR), Pituitary (TPIT), Thyroid (TTRD), Miscellaneous endocrine (TMIE), Hibernating gland (THIB), Miscellaneous glandular (TMIG), Preputial gland (TPPT)
Hematopoietic	Non-thymic lymphoma, generalized (NTYG), Non-thymic lymphoma, localized (NTYL), Thymic lymphoma, generalized (TTYG), Thymic lymphoma, localized (TTYL)
Mesothelium	No match
Nervous	Brain (TBRN), Central nervous system (TCNS), Miscellaneous nervous system (TMIN), Peripheral nervous system (TPNS)
Male Reproductive	Seminal vesicle (TSMV), Testis (TTST), Cowper's gland (TCGL), Epididymis (TEPI), Prostate (TPST)
Female Reproductive	Ovary (TOVE), Mammary Gland (TMGL), Uterus (TUTE), Vagina (TVAG)
Respiratory	Lung (TADN), Miscellaneous lung (TMIL)
Skeletal	Bone (TBON)
Skin	Skin (TSKN)
Soft Tissue	Connective tissue/fibrosarcoma (TCON), Miscellaneous connective tissue (TMIC), Muscle (TMUS)
Special Sense Organ	Harderian gland (THGL)
Urinary	Urinary bladder (TBLA), Kidney (TKID), Miscellaneous urogenital (TMUG)
Non-neoplastic	All Non-tumor codes
Unknown	Cause of death unknown (CDU)

Supplementary Table S5: Janus experiments used for comparisons with IES chronic irradiation data

Supplementary Table S5: Janus experiments used for comparisons with IES chronic irradiation data

	Total Dose (Gy)	Fractions	Fractionation schedule	Dose Rate (cGy/min)	Time Irradiated (min)	Sample Size
Males	0	Variable	Variable	0	0	1724
	9.2	24	1/week	.8516	45	200
	9.6	120	5/week	.006053	1320	80
Females	0	Variable	Variable	0	0	2626
	.43	1	N/A	2.158	20	350
	7.74	24	1/week	.7164	45	367

Supplementary Figure S2 – CoxPH model validation for 300 fractions and KM curves showing differences in survival probability based on fraction number for each cause of death

A

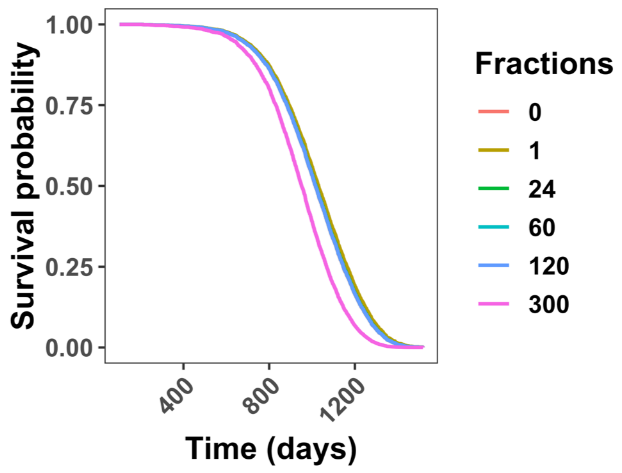

B

Independent Variable	Estimate	Hazard Ratio (95% CI)	P-value
sexM	-0.115	0.892 (0.83, 0.95)	**0.001**
Fractions 1	-0.082	0.921 (0.83, 1.03)	0.134
Fractions 24	-0.014	0.986 (0.88, 1.11)	0.810
Fractions 60	-0.004	0.996 (0.89, 1.11)	0.946
Fractions 120	-0.005	0.995 (0.84, 1.18)	0.953
Fractions 300	0.402	1.49 (1.25, 1.79)	**<0.001**

C

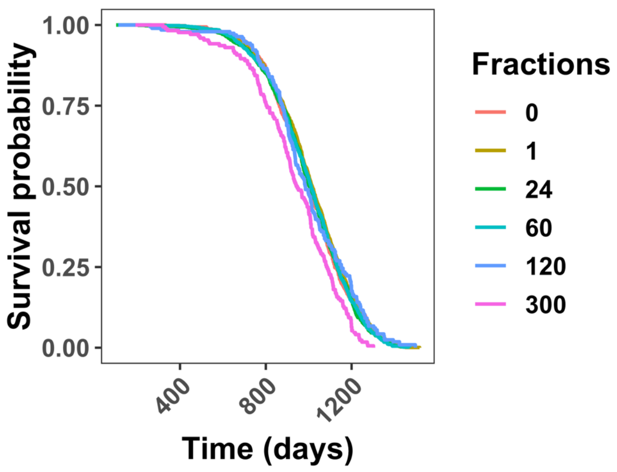

D

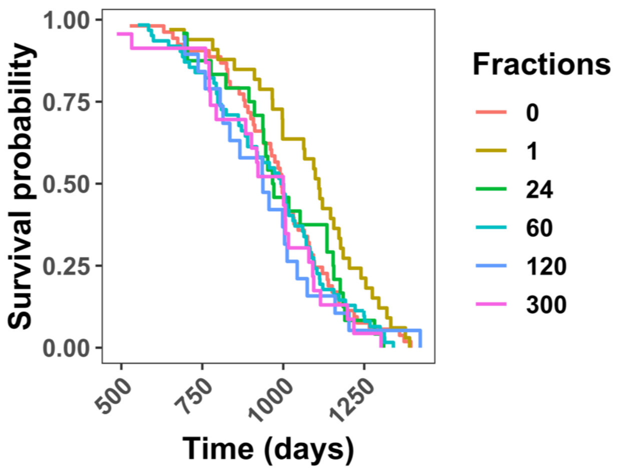

Supplementary Figure S2: Survival probability output from Cox PH model with sex and the number of fractions as a categorical variable control mice **(A/B)**. The predicted outcomes shown **(A)** are for male mice. Kaplan Meier curve for control mice showing differences in survival probability based on the number of fractions used during sham irradiation treatment **(C)**. Kaplan Meier curves showing how the number of fractions impacts survival in control male mice that died of solid tumors (excluding lung tumors) **(D)**, lung tumors **(E)**, lymphomas **(F)**, non-tumor diseases **(G)**, and CDU **(H).**

Supplementary Figure S2 – CoxPH model validation for 300 fractions and KM curves showing differences in survival probability based on fraction number of each cause of death

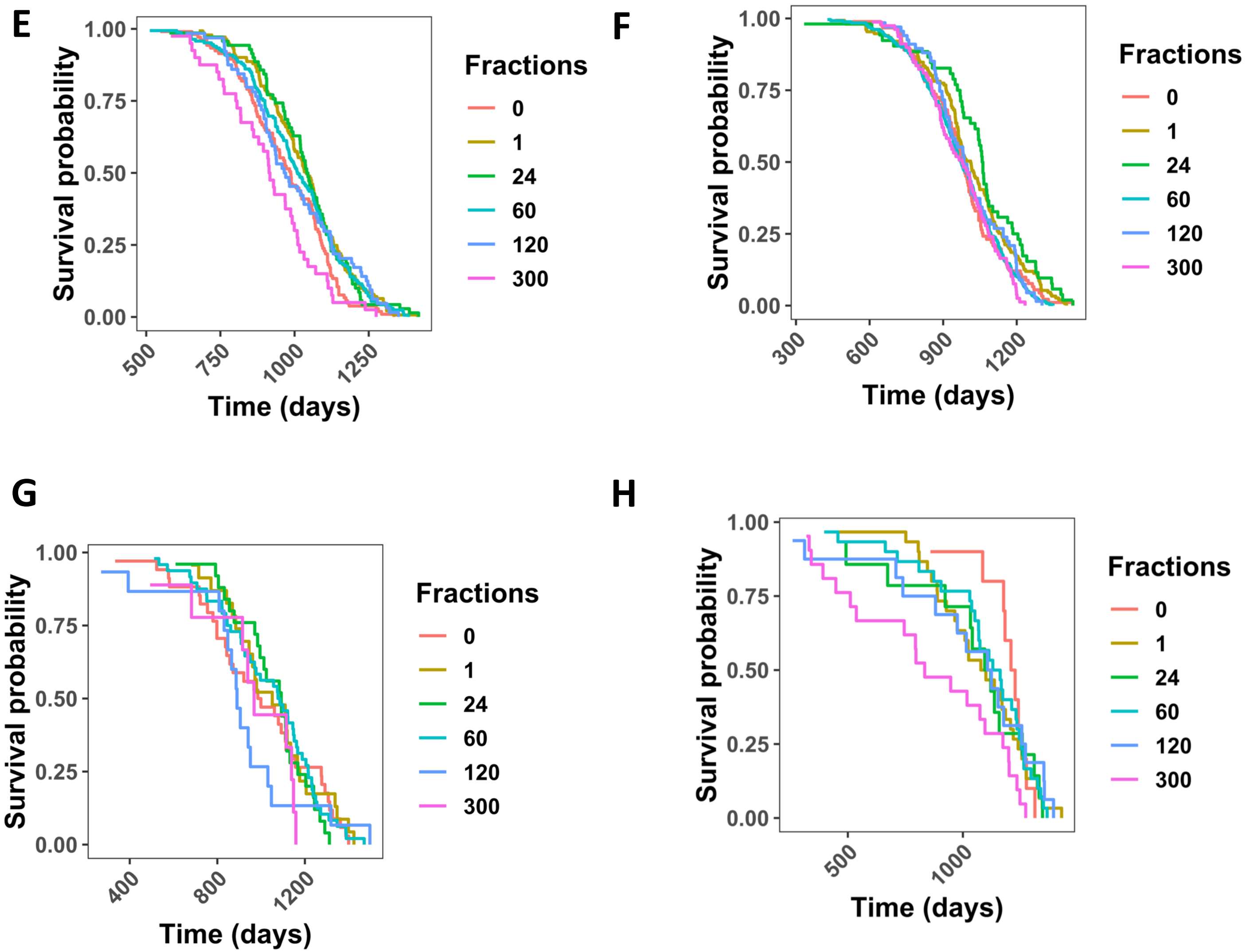

Supplementary Figure S2: Survival probability output from Cox PH model with sex and the number of fractions as a categorical variable control mice **(A/B)**. The predicted outcomes shown **(A)** are for male mice. Kaplan Meier curve for control mice showing differences in survival probability based on the number of fractions used during sham irradiation treatment **(C)**. Kaplan Meier curves showing how the number of fractions impacts survival in control male mice that died of solid tumors (excluding lung tumors) **(D)**, lung tumors **(E)**, lymphomas **(F)**, non-tumor diseases **(G)**, and CDU **(H).**

Supplementary Figure S3: Robustness tests for main model

A

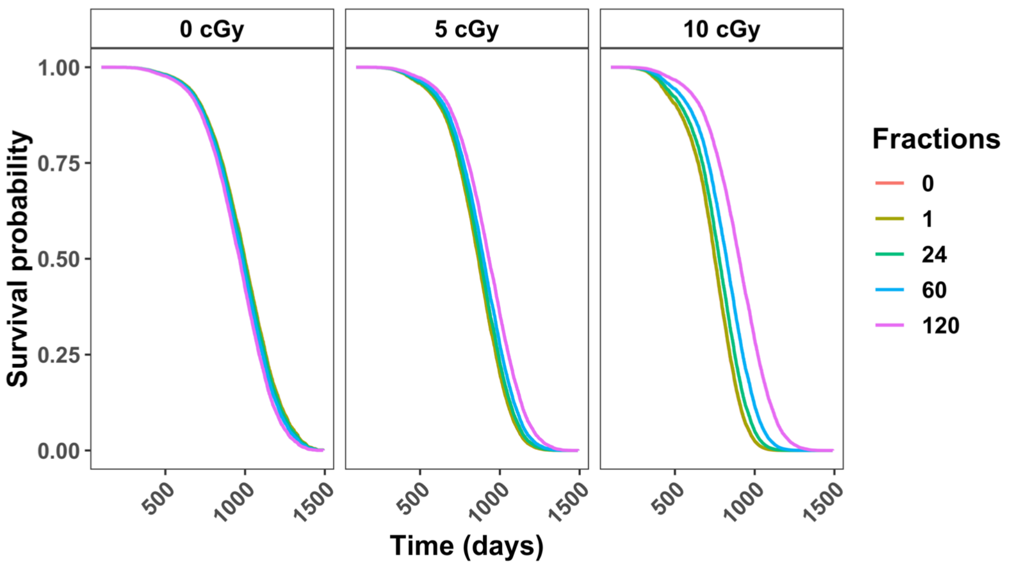

B

Independent Variable	Estimate	Hazard Ratio (95% CI)	P-value
sexM	-0.157	0.85 (0.81, 0.90)	**<0.001**
Fractions	0.002	1.002 (1.002, 1.003)	**0.002**
Total Dose	0.167	1.18 (1.17, 1.19)	**<0.001**
First Irradiated	0	0.9996 (0.9994, 0.9999)	**0.001**
Fractions:Total Dose	-0.001	0.999 (0.999, 0.999)	**<0.001**

Supplementary Figure S3: Robustness testing for gamma irradiation main model. CoxPH model output from the base model with the following changes: **(A/B)** including mice irradiated at all ages, **(C/D)** stratifying by sex, **(E/F)** treating fractions as a categorical variable, **(G/H)** including exact fraction number and age first irradiated for control samples , **(I/J)** including mice with total dose received in 300 fractions, **(K/L)** including mice with total dose received in 300 fractions and fractions treated as a categorical variable, and (M/N) including an interaction term between total dose and sex.. The graphs represent predicted outcomes for female mice first irradiated at 120 days.

Supplementary Figure S3: Robustness tests for main model

C

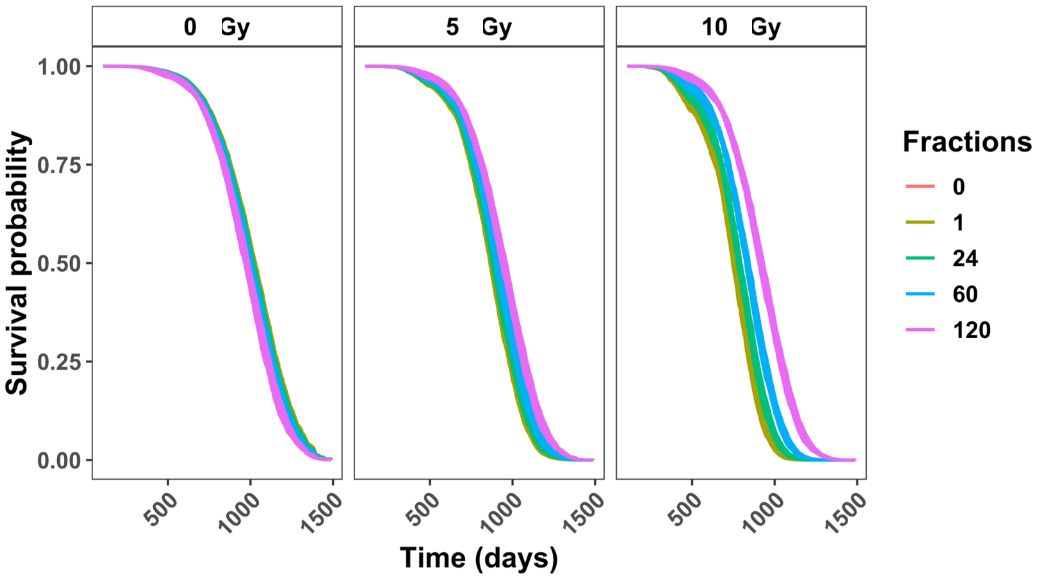

D

Independent Variable	Estimate	Hazard Ratio (95% CI)	P-value
Fractions	0.002	1.002 (1.001, 1.003)	**0.001**
Total Dose	0.169	1.18 (1.18, 1.19)	**<0.001**
First Irradiated	-0.002	0.998 (0.996, 1.0004)	0.124
Fractions:Total Dose	-0.001	0.999 (0.999, 0.999)	**<0.001**

Supplementary Figure S3: Robustness testing for gamma irradiation main model. CoxPH model output from the base model with the following changes: **(A/B)** including mice irradiated at all ages, **(C/D)** stratifying by sex, **(E/F)** treating fractions as a categorical variable, **(G/H)** including exact fraction number and age first irradiated for control samples , **(I/J)** including mice with total dose received in 300 fractions, **(K/L)** including mice with total dose received in 300 fractions and fractions treated as a categorical variable, and (M/N) including an interaction term between total dose and sex.. The graphs represent predicted outcomes for female mice first irradiated at 120 days.

Supplementary Figure S3: Robustness tests for main model

E

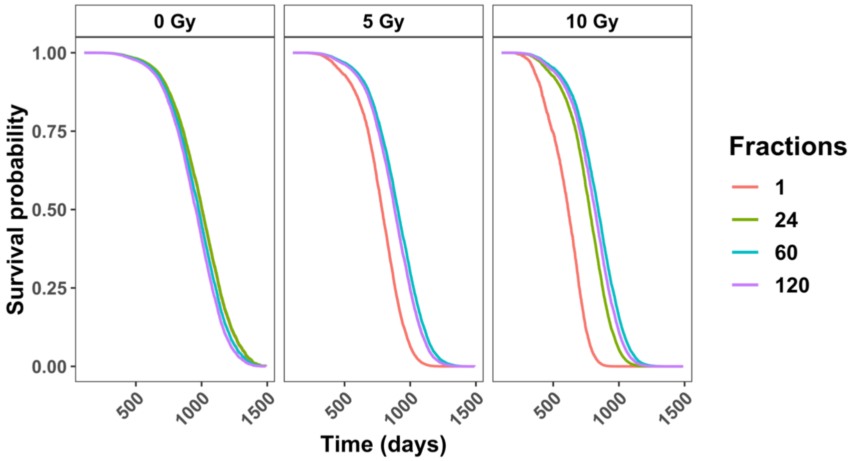

F

Independent Variable	Estimate	Hazard Ratio (95% CI)	P-value
Sex	-0.203	0.82 (0.77, 0.86)	**<0.001**
Fractions 24	-0.015	0.99 (0.88, 1.10)	0.79
Fractions 60	0.143	1.15 (1.04, 1.28)	**0.007**
Fractions 120	0.27	1.31 (1.094, 1.57)	**0.003**
Total Dose	0.278	1.32 (1.228, 1.42)	**<0.001**
First Irradiated	-0.007	0.99 (0.991, 0.996)	**<0.001**
Fractions 24: Total Dose	-0.132	0.88 (0.82, 0.94)	**<0.001**
Fractions 60: Total Dose	-0.193	0.82 (0.77, 0.89)	**<0.001**
Fractions 120: Total Dose	-0.188	0.83 (0.77, 0.89)	**<0.001**

Supplementary Figure S3: Robustness testing for gamma irradiation main model. CoxPH model output from the base model with the following changes: **(A/B)** including mice irradiated at all ages, **(C/D)** stratifying by sex, **(E/F)** treating fractions as a categorical variable, **(G/H)** including exact fraction number and age first irradiated for control samples , **(I/J)** including mice with total dose received in 300 fractions, **(K/L)** including mice with total dose received in 300 fractions and fractions treated as a categorical variable, and (M/N) including an interaction term between total dose and sex.. The graphs represent predicted outcomes for female mice first irradiated at 120 days.

Supplementary Figure S3: Robustness tests for main model

G

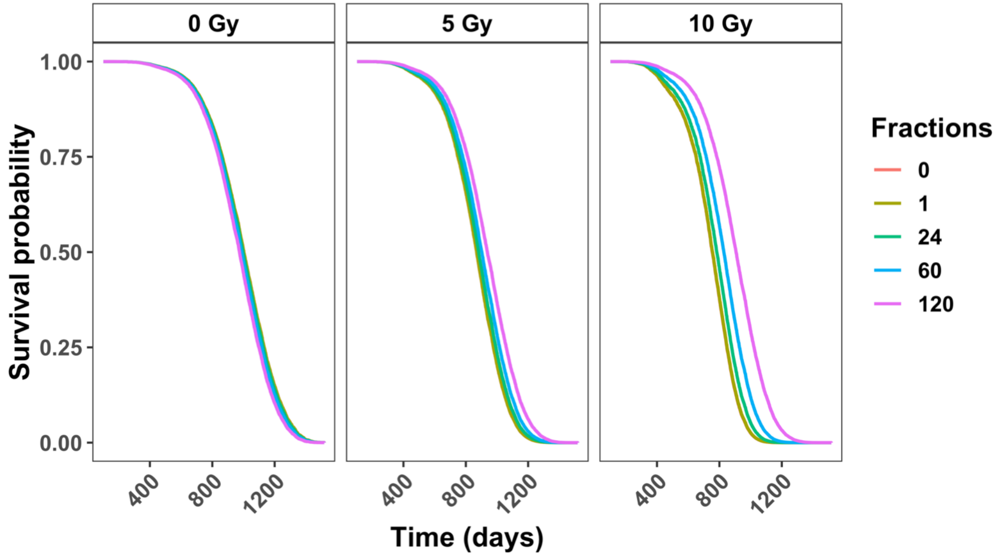

H

Independent Variable	Estimate	Hazard Ratio (95% CI)	P-value
SexM	-0.149	0.86 (0.83, 0.90)	**<0.001**
Total Dose	0.001	1.001 (1.001, 1.002)	**<0.001**
First Irradiated	0.168	1.18 (1.18, 1.19)	**<0.001**
Fractions:Total Dose	-0.002	0.998 (0.996, 0.9996)	**0.017**
Fractions	-0.001	0.999 (0.9990, 0.9991)	**<0.001**

Supplementary Figure S3: Robustness testing for gamma irradiation main model. CoxPH model output from the base model with the following changes: **(A/B)** including mice irradiated at all ages, **(C/D)** stratifying by sex, **(E/F)** treating fractions as a categorical variable, **(G/H)** including exact fraction number and age first irradiated for control samples , **(I/J)** including mice with total dose received in 300 fractions, **(K/L)** including mice with total dose received in 300 fractions and fractions treated as a categorical variable, and (M/N) including an interaction term between total dose and sex.. The graphs represent predicted outcomes for female mice first irradiated at 120 days.

Supplementary Figure S3: Robustness tests for main model

I

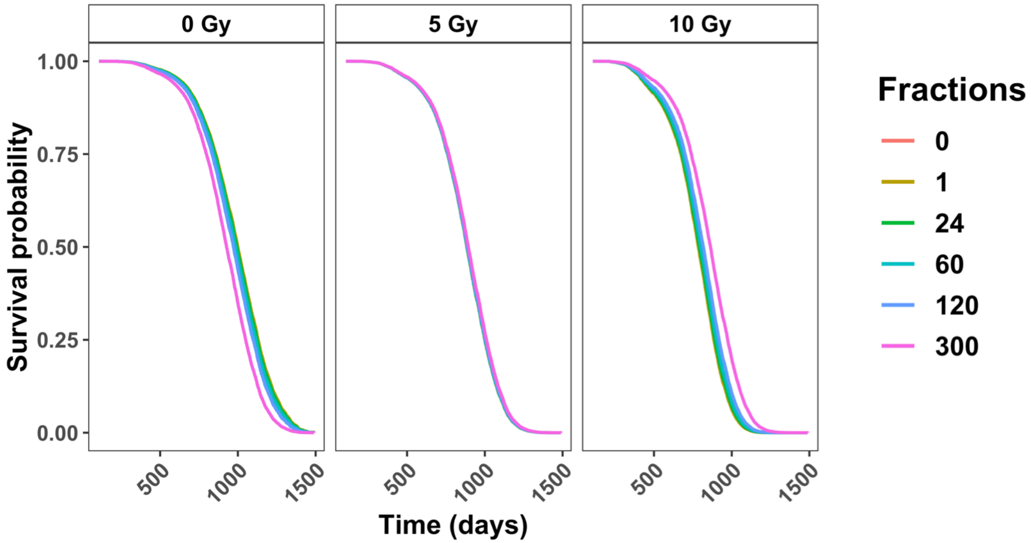

J

Independent Variable (reference)	Estimate	Hazard Ratio (95% CI)	P-value
SexM	-0.182	0.834 (0.79, 0.88)	**<0.001**
Total Dose	0.001	1.0014 (1.007, 1.0020)	**<0.001**
First Irradiated	0.138	1.15 (1.14, 1.15)	**<0.001**
Fractions:Total Dose	-0.002	0.996 (0.9955, 0.9997)	**0.0221**
Fractions	-3.16E-04	0.9997 (0.9996, 0.9997)	**<0.001**

Supplementary Figure S3: Robustness testing for gamma irradiation main model. CoxPH model output from the base model with the following changes: **(A/B)** including mice irradiated at all ages, **(C/D)** stratifying by sex, **(E/F)** treating fractions as a categorical variable, **(G/H)** including exact fraction number and age first irradiated for control samples , **(I/J)** including mice with total dose received in 300 fractions, **(K/L)** including mice with total dose received in 300 fractions and fractions treated as a categorical variable, and (M/N) including an interaction term between total dose and sex.. The graphs represent predicted outcomes for female mice first irradiated at 120 days.

Supplementary Figure S3: Robustness tests for main model

K

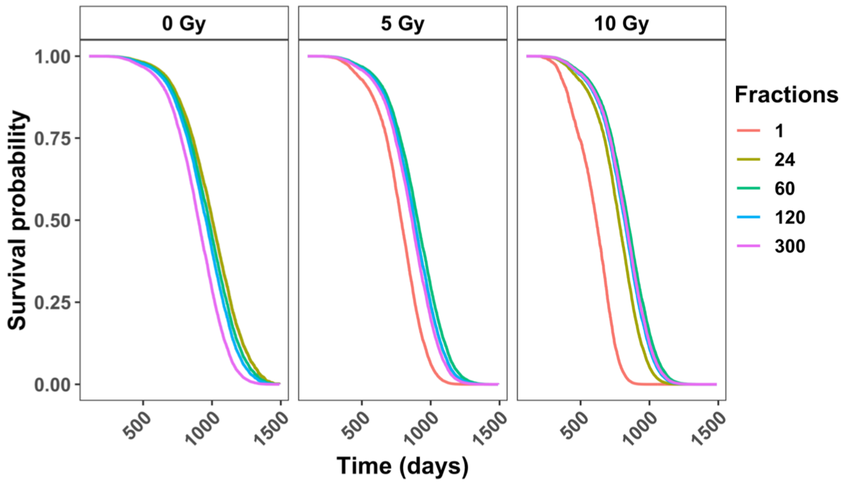

L

Independent Variable (reference)	Estimate	Hazard Ratio (95% CI)	P-value
SexM	-0.203	0.817 (0.77, 0.86)	**<0.001**
Fractions 24	-0.008	0.992 (0.89, 1.11)	0.885
Fractions 60	0.145	1.16 (1.04, 1.28)	**0.0059**
Fractions 120	0.270	1.31 (1.09, 1.57)	**0.0035**
Fractions 300	0.59	1.81 (1.46, 2.25)	**<0.001**
Total Dose	0.279	1.32 (1.23, 1.42)	**<0.001**
First Irradiated	-0.007	0.994 (0.991, 0.996)	**<0.001**
Fractions 24: Total Dose	-0.133	0.88 (0.81, 0.94)	**0.0003**
Fractions 60: Total Dose	-0.194	0.82 (0.77, 0.89)	**<0.001**
Fractions 120: Total Dose	-0.189	0.83 (0.77, 0.89)	**<0.001**
Fractions 300: Total Dose	-0.227	0.80 (0.74, 0.86)	**<0.001**

Supplementary Figure S3: Robustness testing for gamma irradiation main model. CoxPH model output from the base model with the following changes: **(A/B)** including mice irradiated at all ages, **(C/D)** stratifying by sex, **(E/F)** treating fractions as a categorical variable, **(G/H)** including exact fraction number and age first irradiated for control samples , **(I/J)** including mice with total dose received in 300 fractions, **(K/L)** including mice with total dose received in 300 fractions and fractions treated as a categorical variable, and (M/N) including an interaction term between total dose and sex.. The graphs represent predicted outcomes for female mice first irradiated at 120 days.

Supplementary Figure S3: Robustness tests for main model

M

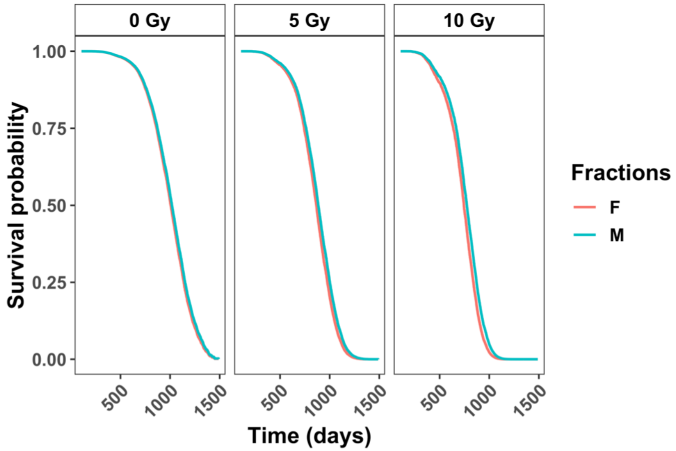

N

Independent Variable	Estimate	Hazard Ratio (95% CI)	P-value
SexM	-0.07	0.93 (0.87588, 0.99347)	**0.03**
Total Dose	17.4	3.77E7 (1.79E, 7.95E7)	**0**
Fractions	0.002	1.0015 (1.07, 1.003)	**0.007**
First Irrad	-0.002	0.998(0.996, 1.0003)	0.102
SexM:Total Dose	-1.552	0.21 (0.122, 0.367)	**0**
Total Dose:Fractions	-0.107	0.898 (0.88, 0.91)	**0**

Supplementary Figure S3: Robustness testing for gamma irradiation main model. CoxPH model output from the base model with the following changes: **(A/B)** including mice irradiated at all ages, **(C/D)** stratifying by sex, **(E/F)** treating fractions as a categorical variable, **(G/H)** including exact fraction number and age first irradiated for control samples , **(I/J)** including mice with total dose received in 300 fractions, **(K/L)** including mice with total dose received in 300 fractions and fractions treated as a categorical variable, and (M/N) including an interaction term between total dose and sex.. The graphs represent predicted outcomes for female mice first irradiated at 120 days.

Supplementary Figure S4: KM curves for control and gamma irradiated mice to validate Cox Proportional Hazards model

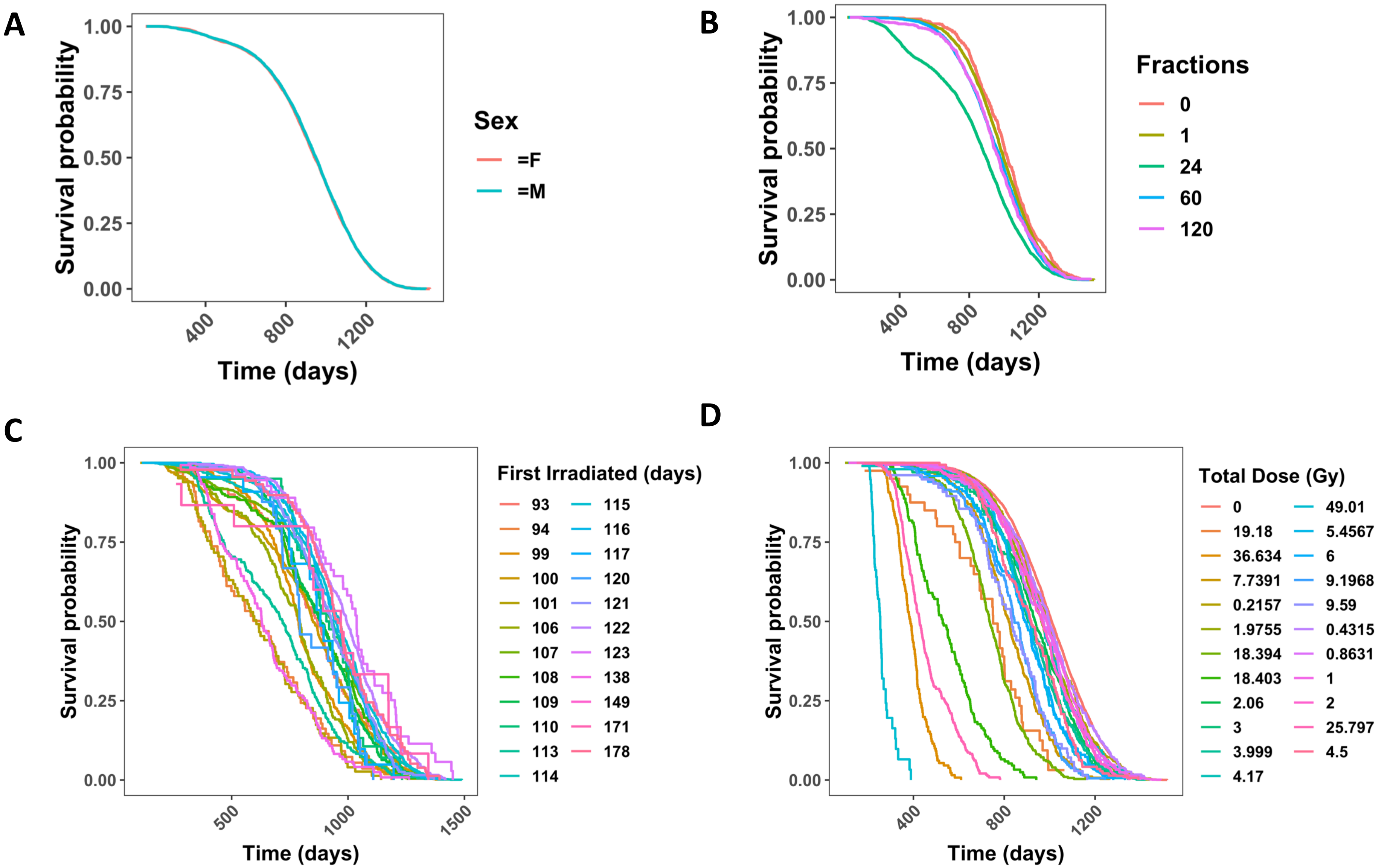

Supplementary Figure S4: Kaplan Meier curves showing survival probably vs. time in gamma irradiated and control mice after the filtering shown in Table 1 and removing mice first irradiated after 500 days. KM curves compare **(A)** sex, **(B)** number of fractions, **(C)** age first irradiated, and **(D)** total dose.

Supplementary Figure S5: Cause specific hazards for lung tumors, non-thymic lymphomas, and tumors (excluding lung tumors)

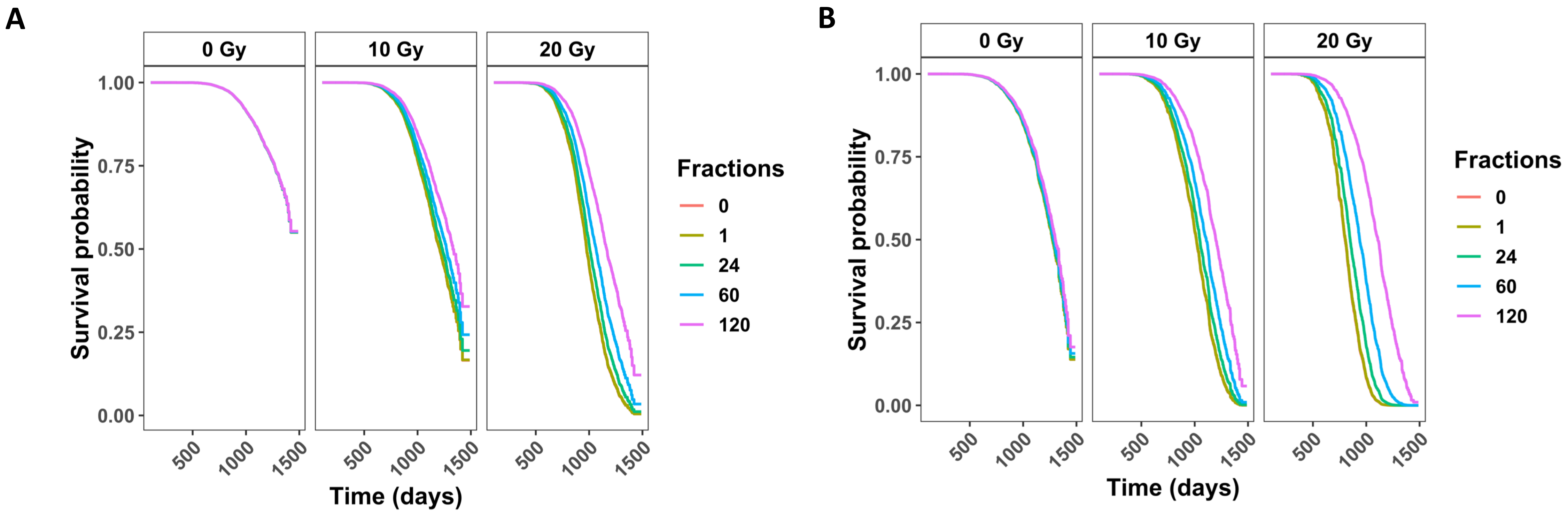

Supplementary Figure S5: Competing risks models for specific causes of death in gamma irradiated mice with age as a time scale and sex, age first irradiated, total dose, fractions, and the interaction between total dose and fractions as independent variables. Survival curves for cause of death being **(A)** lung tumors, **(B)** all tumors (excluding lung tumors), and **(C)** non-thymic lymphomas. The corresponding model output with parameter estimates, hazard ratios with a 95% confidence interval, and p-values are listed in **table 2** for lung tumors and all tumors (excluding lung tumors). **(D)** Model output for non-thymic lymphoma. The graphs represent predicted outcomes for female mice first irradiated at 120 days.

Supplementary Figure S5: Cause specific hazards for lung tumors, non-thymic lymphomas, and tumors (excluding lung tumors)

C

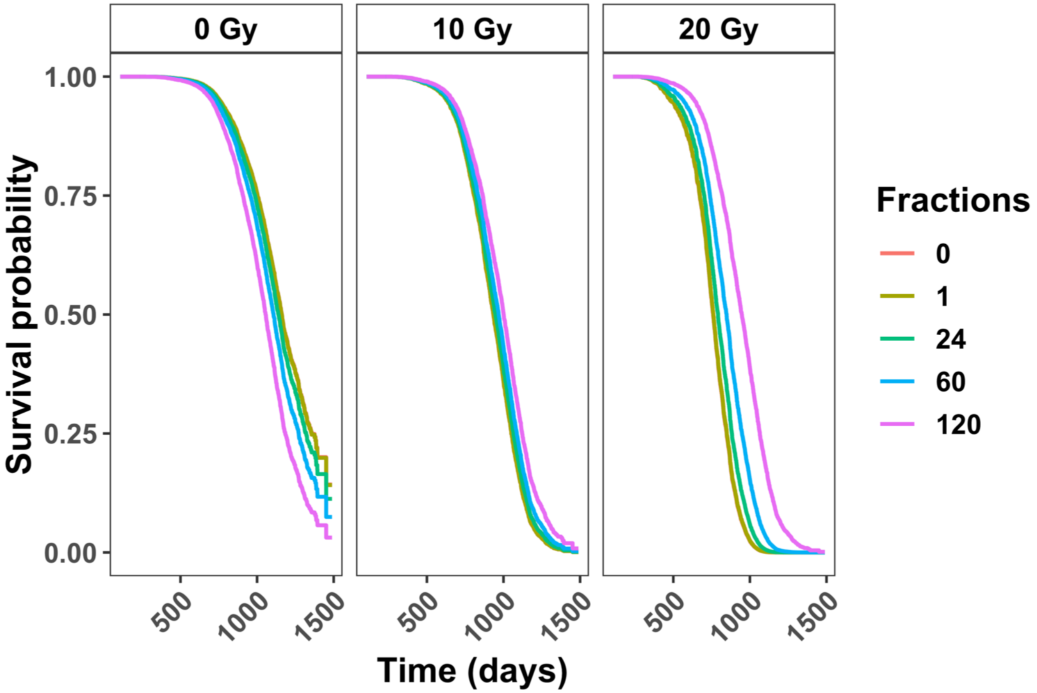

D

Term	Estimate	Hazard Ratio (95%)	P-value
SexM	-0.33	0.72 (0.662, 0.786)	**<0.001**
Fractions	0.005	1.005 (1.003, 1.007)	**<0.001**
Total Dose	0.129	1.14 (1.12, 1.16)	**<0.001**
First Irradiated	-0.005	0.995 (0.991, 0.999)	**0.011**
Fractions:Total Dose	-0.001	0.9992 (0.9990, 0.9995)	**<0.001**

Supplementary Figure S5: Competing risks models for specific causes of death in gamma irradiated mice with age as a time scale and sex, age first irradiated, total dose, fractions, and the interaction between total dose and fractions as independent variables. Survival curves for cause of death being **(A)** lung tumors, **(B)** all tumors (excluding) lung tumors, and **(C)** non-thymic lymphomas. The corresponding model output with parameter estimates, hazard ratios with a 95% confidence interval, and p-values are listed in **table 2** for lung tumors and all tumors (excluding lung tumors). **(D)** Model output for non-thymic lymphoma. The graphs represent predicted outcomes for female mice first irradiated at 120 days.

Supplementary Figure S6: CIF with a dose cutoff of 6Gy

A

Variable	Parameter Estimate	Hazard Ratio (95% CI)	P-value
SexM	-0.423	0.655 (0.57, 0.75)	**<0.001**
Fractions	0.043	1.044 (0.978, 1.113)	0.2
Total Dose	-1.30E-03	0.999 (0.998, 0.999)	**<0.001**
First Irradiated	-3.73E-03	0.996 (0.993, 1.000)	0.07
Fractions:Total Dose	0.001	1.001 (0.999, 1.002)	0.32

B

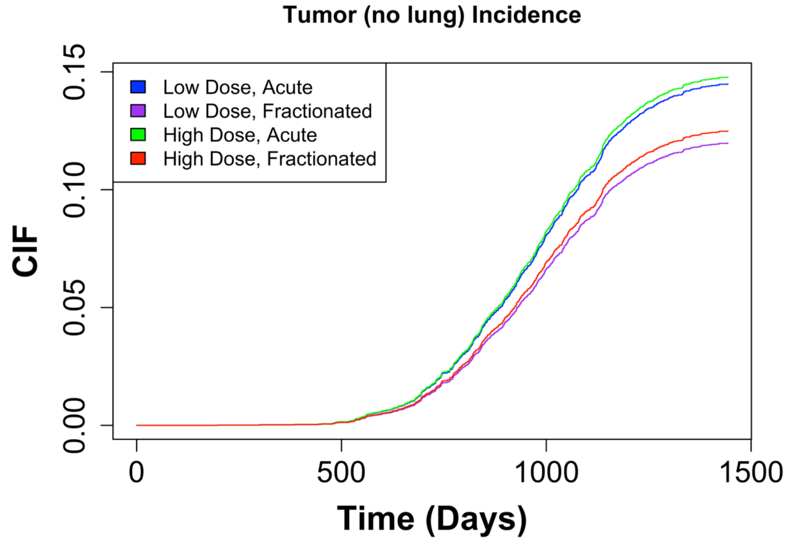

C

Variable	Parameter Estimate	Hazard Ratio (95% CI)	P-value
SexM	1.26	3.524 (3.04, 4.08)	**<0.001**
Fractions	0.016	1.016 (0.954, 1.083)	0.62
Total Dose	2.14E-04	1.000 (1.000, 1.00)	.45
First Irradiated	-3.23E-03	0.997 (0.993, 1.00)	0.094
Fractions:Total Dose	0.001	1.001 (0.999, 1.002)	0.32

D

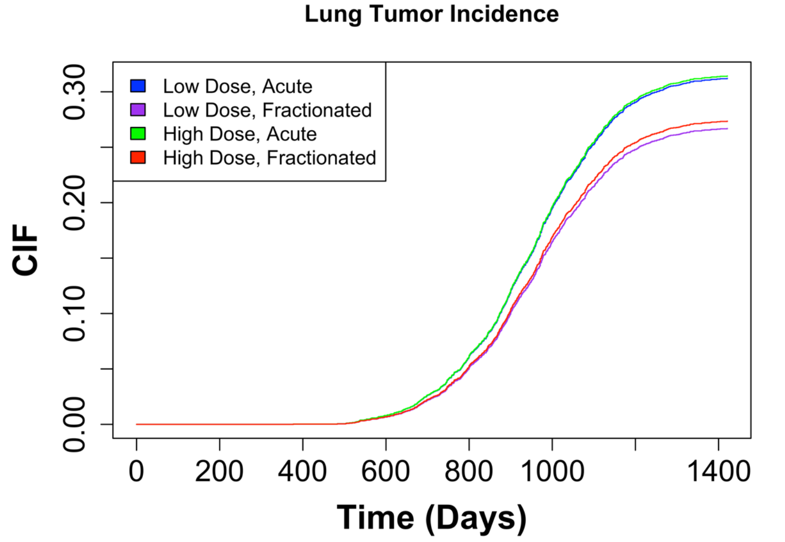

Supplementary Figure S6: CIF regression model output using a total dose cutoff of 6Gy for tumor (no lung) **(A),** lung tumor **(C)**, lymphoma **(E)**, and non-tumor deaths **(G)**. Predicted output from CIF model for tumor (no lung) **(B),** lung tumor **(D)**, lymphoma **(F)**, and non-tumor deaths **(H)**. All predicted outputs represent males first irradiated at 120 days under the following conditions: low dose = 0.1Gy, high dose = 6Gy, acute = 1 fraction, fractionated = 60 fractions.

Supplementary Figure S6: CIF with a dose cutoff of 6Gy

E

Variable	Parameter Estimate	Hazard Ratio (95% CI)	P-value
SexM	-0.276	0.759 (0.69, 0.83)	**<0.001**
Fractions	-0.023	0.977 (0.936, 1.02)	0.29
Total Dose	-2.23E-04	1.000 (0.999, 1.00)	0.36
First Irradiated	6.25E-03	1.006 (1.004, 1.01)	**<0.001**
Fractions:Total Dose	-0.001	0.999 (0.998, 1)	**0.014**

F

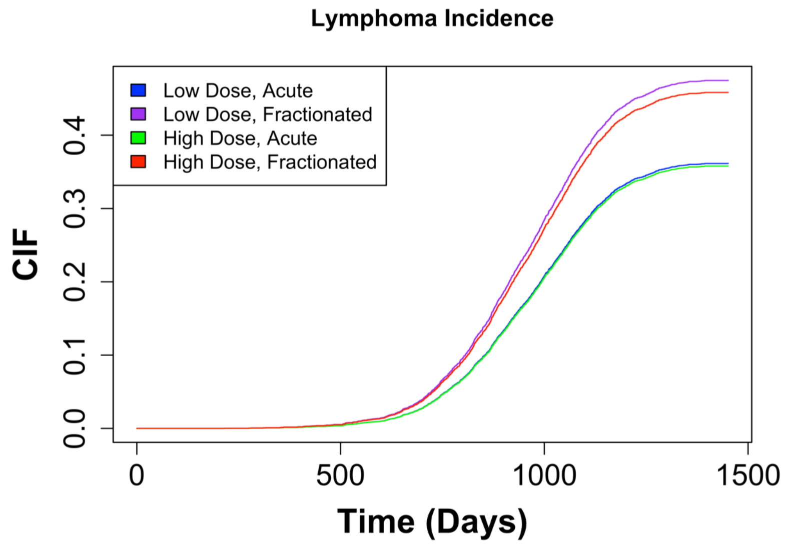

G

Variable	Parameter Estimate	Hazard Ratio (95% CI)	P-value
SexM	-0.342	0.71 (0.608, 0.83)	**<0.001**
Fractions	0.118	1.126 (1.049, 1.207)	**0.001**
Total Dose	0.001	1.001 (1, 1.001)	0.15
First Irradiated	-0.01	0.99 (0.985, 0.995)	**<0.001**
Fractions:Total Dose	0.001	1.001 (0.999, 1.003)	0.28

H

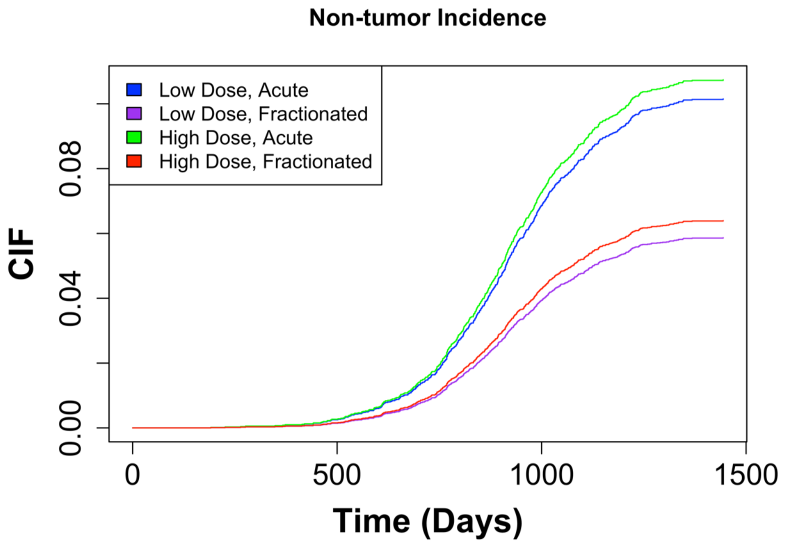

Supplementary Figure S6: CIF regression model output using a total dose cutoff of 6Gy for tumor (no lung) **(A),** lung tumor **(C)**, lymphoma **(E)**, and non-tumor deaths **(G)**. Predicted output from CIF model for tumor (no lung) **(B),** lung tumor **(D)**, lymphoma **(F)**, and non-tumor deaths **(H)**. All predicted outputs represent males first irradiated at 120 days under the following conditions: low dose = 0.1Gy, high dose = 6Gy, acute = 1 fraction, fractionated = 60 fractions.
